# On the Enantioselective HPLC Separation Ability of Sub-2 µm Columns: Chiralpak^®^ IG-U and ID-U

**DOI:** 10.3390/molecules24071287

**Published:** 2019-04-02

**Authors:** Diana Ibrahim, Ashraf Ghanem

**Affiliations:** Chirality Program, Faculty of Science and Technology, University of Canberra, ACT 2601, Australia; diana.ibrahim@canberra.edu.au

**Keywords:** Chiralpak^®^ ID-U, Chiralpak^®^ IG-U, mobile phase modifiers, polar organic and reversed phase modes, sub-2 μm particles

## Abstract

Silica with a particle size of 3–5 µm has been widely used as selector backbone material in 10–25 cm HPLC chiral columns. Yet, with the availability of 1.6 µm particles, shorter, high-efficiency columns practical for minute chiral separations are possible to fabricate. Herein, we investigate the use of two recently commercialized sub-2 µm columns with different substituents. Thus, Chiralpak^®^ IG-U and ID-U were used in HPLC for the fast enantioseparation of a set of drugs. Chiralpak^®^ IG-U [amylose tris (3-chloro-5-methylphenylcarbamate)] has two substituents on the phenyl ring, namely, a withdrawing chlorine group in the third position and a donating group in the fifth position. Chiralpak^®^ ID-U [amylose tris (3-chlorophenylcarbamate)] has only one substituent on the phenyl ring, namely a withdrawing chlorine group. Their applications in three liquid chromatography modes, namely, normal phase, polar organic mode, and reversed phase, were demonstrated. Both columns have similar column parameters (50 mm length, 3 mm internal diameter, and 1.6 µm particle size) with the chiral stationary phase as the only variable. Improved chromatographic enantioresolution was obtained with Chiralpak^®^ ID-U. Amino acids partially separated were reported for the first time under an amylose-based sub-2-micron column.

## 1. Introduction

In nature and chemical systems, enantiomeric distinction and chiral recognition are fundamental occurrences [1]. This phenomenon has had a profound impact on a plethora of scientific fields, though the pharmaceutical industry significantly drives developments in chirotechnologies to cater to the demands of drug discovery [2,3]. There is no option when it comes to chiral considerations; all enantiomers must be tested in isolation of each other before being introduced to the market [3]. As a result, high performance liquid chromatography (HPLC) has emerged as the workhorse for racemate resolution [4]. HPLC enantiomer separation using chiral stationary phases (CSPs) is known to be one of the most convenient and versatile methods for the separation of chiral drugs [4].

In the last few decades, numerous CSPs have been developed and become commercially available [5,6]. CSPs filled in conventional columns of 4.0–4.6 mm internal diameter (i.d.) are the most widely used for analytical scale enantioseparation for industrial applications [5,6]. Nonetheless, conventional chiral columns are expensive; they consume large volumes of hazardous solvents and have long analysis times, and due to the dimensions of these large columns they are of limited throughput [6]. One of the possible solutions to enhance the speed of the analysis is to use columns filled with a CSP of smaller particles (sub-2 µm) and hence a smaller theoretical plates height [7].

Sub-2 µm totally porous particles can be used to speed up analysis without loss in efficiency, as the optimal flow rate is inversely proportional to particle diameter [8]. The main limitation of using totally porous particles is the induction of high back pressure across the column induced by the friction of the mobile phase percolating through the particles generating heat, which hinders their usage within conventional HPLC systems [9]. Studies suggest that small i.d. columns can be used to minimize the frictional heating effect since heat dissipation is faster within such a narrow-bore column compared to conventional 4.6 mm i.d. columns [10]. Narrow-bore columns have a lower internal volume (2.1 mm i.d.) than the standard HPLC columns and thus achieve fast analysis [10,11]. They operate at lower flow rates (0.1–0.5 mL/min) with much reduced peak volumes, resulting in reduced mobile phase consumption and increased sensitivity [11,12].

Mobile phases can be modified to achieve higher enantioselective separation of racemates via improvement of complementary interactions between functional groups on the chiral selector and the analyte structure [13]. Pirkle and Welch have studied modifier effects on chiral selectivity and found that the influence of the mobile phase modifier was dependent upon the analyte structure [13,14,15]. Tambute and co-workers have also examined the use of modifiers and concluded that selectivity in their system depends on the steric hindrance of the alcohol modifier [14,15,16]. Researchers believe that the mobile-phase modifiers not only compete for chiral bonding sites with chiral solutes but can also alter the steric environment of the chiral grooves on the CSP by binding to the achiral sites at or close to the groove [13,17]. Enantioselective resolution is mainly due to the overall combination of all types of bonding [18]. Thus, not only the steric but also the substitutes of a certain chiral compound and the CSP should be taken into consideration to elucidate chiral recognition mechanisms [19].

Here we evaluate and compare the enantiorecognition abilities of two amylose-based sub-2 µm CSPs towards 28 compounds, as they differ in the substituents on the phenyl ring. Recently commercialized Chiralpak^®^ IG-U [amylose tris (3-chloro-5-methylphenylcarbamate)] possesses an extra donating methyl group in the fifth position compared to the prototype Chiralpak^®^ ID-U [amylose tris (3-chlorophenylcarbamate)]. This investigation was performed using an operational instrument at an HPLC system pressure of 500 bar at which frictional heating is not very significant. Hence, thermal gradients inside the column were not expected to affect the efficiency.

## 2. Experimental

### 2.1. Instrumentation

The mobile phase for the HPLC was filtered through a Millipore membrane filter (0.2 µm) and degassed before use. The HPLC system consisted of a Waters binary pump, Model 1525, (Milford, MA, USA), equipped with a dual wavelength absorbance detector, Model 2487, an autosampler, Model 717 plus, and an optical rotation detector (JM Science Inc., Grand Island, NY, USA) operating at room temperature. The UV-detector was set at 254 nm. Chiralpak^®^ IG-U and ID-U (50 mm column length, 3.0 mm i.d, and 1.6 µm silica gel) were supplied by Daicel (Tokyo, Japan).

### 2.2. Chemicals and Reagents

All compounds and solvents (HPLC grade) were purchased from Sigma-Aldrich (St. Louis, MO, USA). The choice of compounds was arbitrary and guided by preliminary investigations. The compounds were, namely: beta-blockers (propranolol and atenolol), alpha-blockers (naftopidil), anti-inflammatory compounds (carprofen, naproxen, flurbiprofen, ketoprofen, and indoprofen), anticancers (ifosfamide), sedative hypnotics (aminoglutethimide), antiarrhythmic drugs (tocainide), norepinephrine-dopamine reuptake inhibitors (nomifensine), catecholamines (normetanephrine and epinephrine), antihistamines (chlorpheniramine), flavonoids (flavanone and 6-hydroxyflavanone), miscellaneous (1-acenaphthenol, 1-indanol, 4-hydroxy-3-methoxymandelic acid, propafenone HCL, cizolirtine, and 1-phenyl-2,2,2-trifluoroethanol), amino acids (glutamic acid, tyrosine, and phenylalanine) and antifungals (miconazole and sulconazole).

### 2.3. Procedures

Mobile phases were filtered through a membrane Sartorius Minisart RC 15 0.2 µm pore size filter (Goettingen, Germany), further used for analysis without dilution, and degassed before use. The chromatographic measurements were performed at a flow rate of 0.5 mL/min at a temperature of 25 °C. All measurements were performed in triplicate with an injection volume of 1 µL. Stock solutions of samples were prepared at a concentration of 1 mg/mL using HPLC-grade 2-propanol as a solvent.

## 3. Results and Discussion

**Figure 1:**
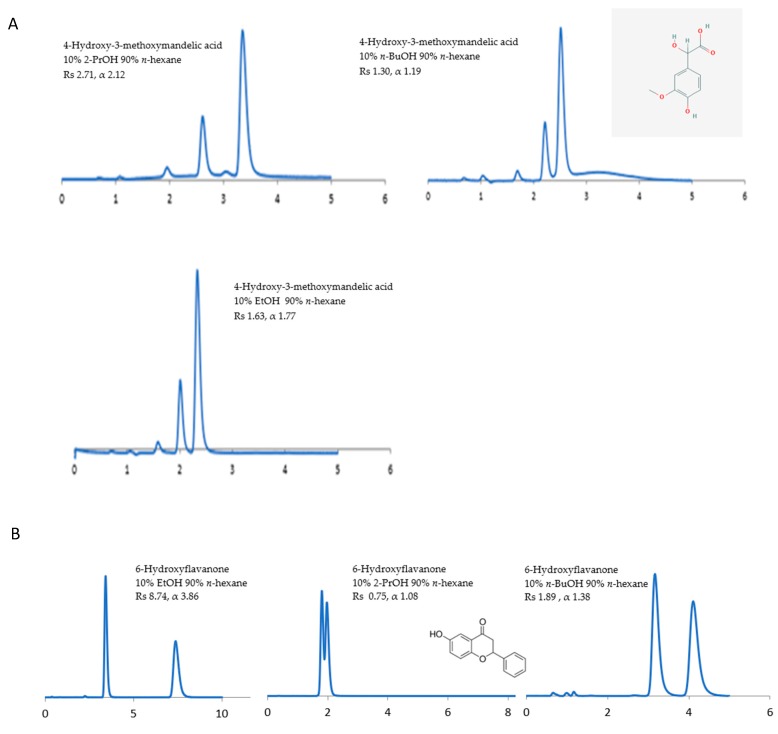
The effect of different alcohol modifiers: 2-propanol (2-PrOH), ethanol (EtOH), and *n*-butanol (*n*-BuOH) on enantioselectivity under two sub-2-micron chiral stationary phases. (**A**) The effect of different alcohol modifiers on 4-hydroxy-3-methoxymandelic acid using Chiralpak^®^ ID-U. (**B**) The effect of different alcohol modifiers on 6-hydroxyflavanone using Chiralpak^®^ IG-U.

**Figure 2:**
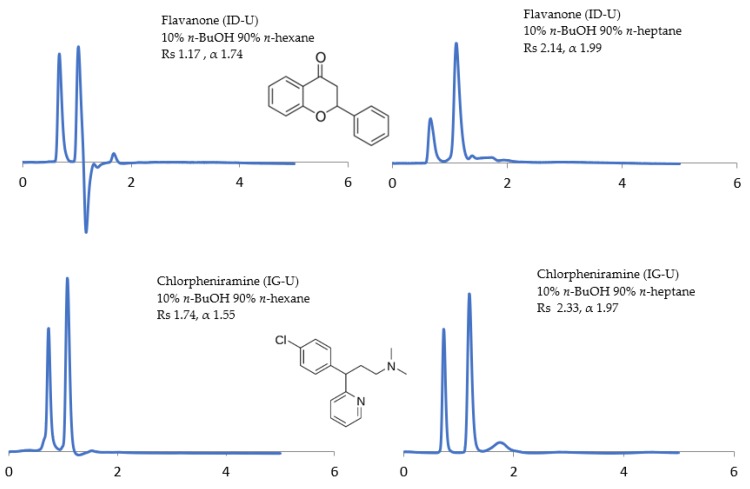
Effect of *n*-hexane versus *n-*heptane on resolution (Rs) and separation factor (α) using Chiralpak^®^ IG-U and ID-U.

**Figure 3:**
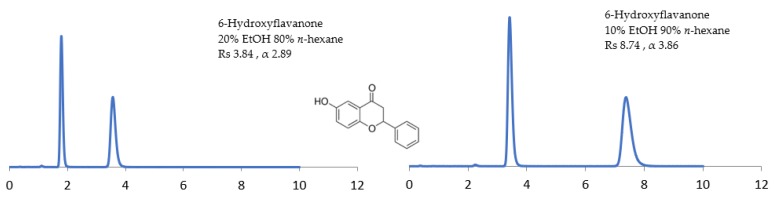
The effect of decreasing the alcohol percentage on chiral selectivity and time taken for the baseline separation of 6-hydroxyflavanone.

**Figure 4:**
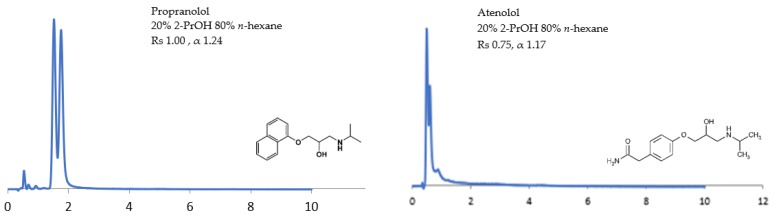
The effect of 20% 2-propanol (2-PrOH) on the stereoselective interactions of β-blockers.

**Figure 5:**
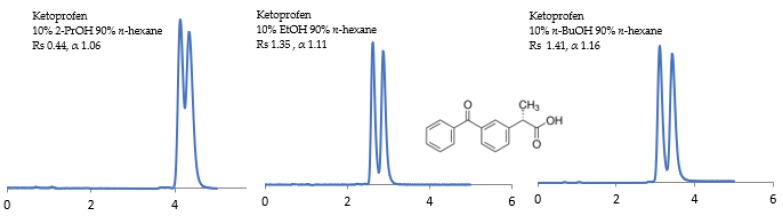
Effect of different alcohol modifiers with *n*-hexane on resolution (Rs) and separation factor (α) of ketoprofen. Ketoprofen expressed an increasingly enhanced Rs and α in the order of 2-propanol (2-PrOH) to ethanol (EtOH) to *n*-butanol (*n*-BuOH).

**Figure 6:**
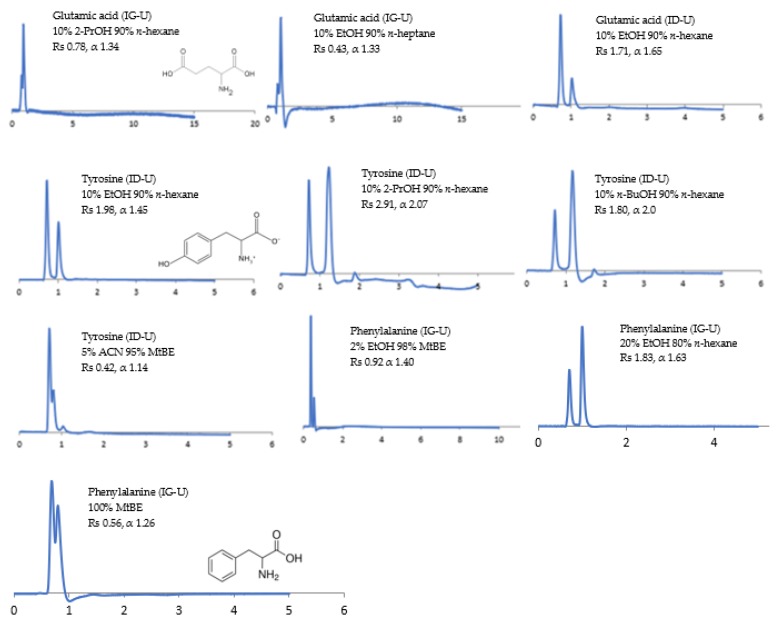
Enantioselectivity of three amino acids under different mobile phase compositions.

**Figure 7:**
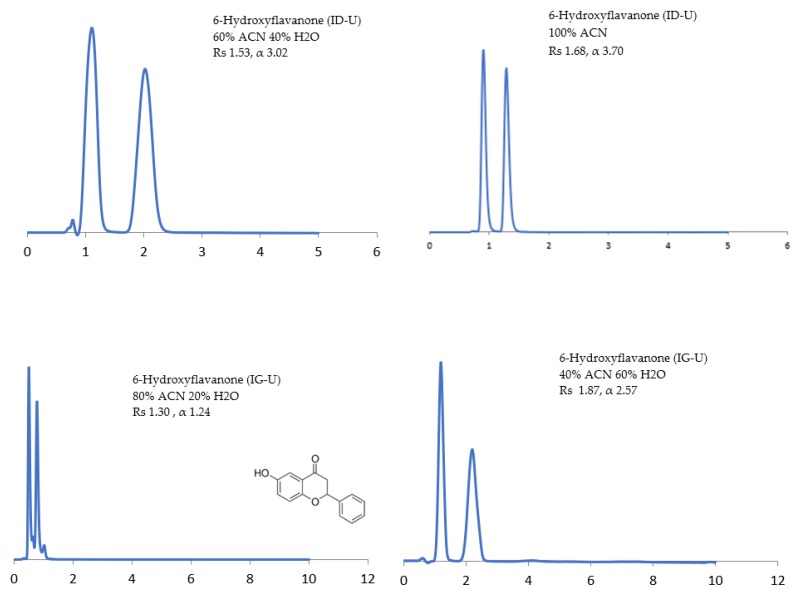
Enantioseparation under organic-aqueous conditions and the effect of water in acetonitrile (ACN) mobile phase on resolution (Rs) and separation factor (α) of 6-hydroxyflavanone.

The potential of the sub-2 μm CSPs to separate the racemic compounds listed above under normal-phase, reversed-phase, and polar organic solvents have been investigated. The influence of the mobile phase composition on the separation (α), resolution (Rs), and retention time (RT) of enantiomers has been examined using (1) non-polar solvents (n-alkanes) containing a polar alcohol modifier, namely, ethanol (EtOH), 2-propanol (2-PrOH), and *n*-butanol (*n*-BuOH), and (2) polar solvents, namely, methyl tert-butyl ether (MtBE), acetonitrile (ACN), 1,4-dioxane, and dichloromethane (DCM). The CSP structural differences under different mobile phase conditions are reflected in some selected chromatograms shown in Figure 1, Figure 2, Figure 3, Figure 4, Figure 5, Figure 6 and Figure 7.

### 3.1. Enantioselectivity under Non-Polar Solvents Containing an Alcohol Polar Modifier

The initial mobile phase composition of *n*-hexane/alcohol modifier (90/10, *v/v*) was prepared. Out of the three alcohol modifiers tested, *n*-BuOH showed the lowest enantioselectivity in both tested CSPs, namely, Chiralpak^®^ IG-U and ID-U. This might be due to the difference in the steric bulkiness around the hydroxyl moiety contained in the mobile phase modifier [15,16,17,18]. Conversely, EtOH afforded better enantioselectivity for both CSPs. Upon replacement of EtOH with bulkier *n*-BuOH, the competition for hydrogen-bonding sites on these CSPs becomes weaker. This might be due to the fact that lower alcohols such as EtOH are unlike bulkier alcohols and could diffuse more easily into well-defined grooves of the CSP. Thus, more stable diastereomeric complexes with the enantiomers could be formed, consequently resulting in higher Rs and α value [15,17,18,19,20,21]. Of particular interest is that ifosfamide and glutamic acid were only separated under *n*-hexane/EtOH on Chiralpak^®^ ID-U.

In a few cases, such as with 4-hydroxy-3-methoxymandelic acid, 1-acenaphthenol, 1-indanol, and propafenone HCL, the use of 2-PrOH as an alcohol modifier afforded superior Rs and α on Chiralpak^®^ ID-U. By contrast, these compounds expressed the best Rs and α using EtOH on Chiralpak^®^ IG-U. For example, 4-hydroxy-3-methoxymandelic acid expressed a superior Rs of 2.71 and α of 2.12 on Chiralpak^®^ ID-U (Figure 1A) under *n*-hexane/2-PrOH (90/10, *v/v*) compared to Rs 1.63 and α 1.77 under *n*-hexane/EtOH. Chiralpak^®^ IG-U expressed the best Rs 8.74 and α 3.86 under *n*-hexane/EtOH compared to Rs 0.75 and α 1.08 under *n*-hexane/2-PrOH (Figure 1B). In particular, 1-phenyl-2,2,2-trifluoroethanol with Rs 2.38 and α 3.90, cizolirtine with Rs 5.27 and α 3.39, and naftopidil with Rs 1.75 and α 1.95 were only successfully separated under *n*-hexane/EtOH (90/10, *v/v*) using Chiralpak^®^ IG-U.

The results indicate that the different structural features of the CSP, combined with the incorporation of the alcoholic modifiers of different sizes/shapes, ultimately results in a different stereo environment of the chiral cavities in the CSP, yielding different chiral selectivities [21,22,23,24,25].

Previous studies have showed improvements in selectivity with *n-*heptane over *n-*hexane [26,27]. Therefore, in the current study, *n*-hexane was replaced with *n*-heptane. For example, flavonoids (6-hydroxyflavanone and flavanone) using Chiralpak^®^ ID-U showed an enhanced Rs and α under *n*-heptane. As shown in Figure 2, flavanone showed an enhanced Rs 2.14 and α 1.99 under *n*-heptane/*n*-BuOH (90/10, *v/v*) compared to Rs 1.17 and α 1.74 under *n*-hexane/*n*-BuOH (90/10, *v/v*). The effect of different alcohol modifiers used on Chiralpak^®^ IG-U expressed a range of results in the transition between *n-*hexane to *n*-heptane. For example, chlorpheniramine showed an enhanced Rs which increased from 1.74 to 2.33 and an α which increased from 1.55 to 1.97 using *n*-heptane.

### 3.2. The Effect of Alcohol Modifier Percentage on Enantioselectivity

The composition of the alcohol modifier in the mobile phase was evaluated at 10%–40% *v*. Increasing the composition of the alcohol modifier increases the strength of the mobile phase (the ability of compounds to elute quicker from the column) and hence the RT will consequently be reduced (at the expense of Rs and α, however) [22,23,24,25]. For example, 6-hydroxyflavanone achieved baseline separation in 4 min with Rs 3.85 and α 2.89 under 20% EtOH compared to 8 min with Rs 8.74 and α 3.86 with 10% EtOH on Chiralpak^®^ IG-U (Figure 3). These results indicate that alcohol molecules compete with the analytes for achiral and chiral adsorption sites on the CSP. Thus, RT, α and Rs are altered by changes in the concentration of alcohol [22,23,24].

### 3.3. Effect of the Structure of Analytes on Enantiomeric Separation

It is known that the alcohol modifiers used in the normal-phase mode have a profound influence on the chiral selectivity of CSPs. Therefore, gaining structural information regarding the CSPs in contact with mobile phases containing different alcohol modifiers would be of interest. Polar and π-π interactions between the CSP phenyl groups and the functional group of the solute may also play a role in chiral recognition [21,22,28]. It has been hypothesized that with an increase in the mobile phase polarity, the strength of the hydrogen bonds between the analytes and the CSP decreases and the solubility of the analytes in the mobile phase increases [26,27,28,29]. Moreover, it is possible that some alcohol molecules are associated with the CSP and cause swelling of the column, which leads to opening of the chiral cavities. Thus, the inclusion interactions of the enantiomers are diminished and RT is decreased [26,27,28,29].

#### 3.3.1. β-Blockers

β-blockers are hydroxylamines with functional groups bearing secondary amines or N-isopropyl amines. These drugs also contain aromatic rings with different substituent moieties. The OH and NH groups and an oxygen atom in the model examples of β-blockers studied (propranolol and atenolol) are functional groups which are available to take part in hydrogen bonding with the C=O and NH groups of the CSPs [14,27]. As shown in Figure 4, under 20% 2-PrOH, atenolol has the lowest Rs of 0.75 and α of 1.17. By contrast, propranolol has the largest Rs of 1.00 and α of 1.24. A possible explanation for these results could be that the naphthalene ring of propranolol can form stronger interactions with the CSP [14]. On the other hand, the amide group of atenolol could compete with the groups on the CSP for bonding sites, causing low stereoselective interactions. Furthermore, the CSP-substituted phenyl ring interaction might also be important where the pronounced steric effect could be close to the analyte chiral center, resulting in poor chiral discrimination of atenolol [19]. Both groups adjacent to the chiral centres and the substituent groups on the phenyl rings could contribute to an enhanced separation result [28,29,30,31,32].

#### 3.3.2. Anti-Inflammatory

Out of the four profens used in this study (flurbiprofen, ibuprofen, naproxen, and ketoprofen), ibuprofen and naproxen achieved the lowest Rs and α values under normal phase conditions (an alkane/alcohol modifier). However, they expressed much higher enantio-separation under reversed phase conditions (100% ACN, *v*), (ACN/H_2_0, 60/40, *v/v*). On the other hand, flurbiprofen and ketoprofen expressed a higher Rs under normal phase conditions. In particular, Chiralpak^®^ ID-U showed significantly higher enantio-selectivity values for the tested profens. This column was able to partially separate all tested profens while Chiralpak^®^ IG-U was less effective in the chiral separation of ibuprofen and ketoprofen. Contrary to the literature, as shown in Figure 5, the order of increasing enantioselectivity is 2-PrOH < EtOH < *n*-BuOH. It is hypothesized that hydrogen-bonding might be a predominant factor between the solutes and the CSPs [20,33,34].

#### 3.3.3. Amino Acids

Amino acids (H_2_NCHRCOOH) have three main groups: the carboxyl group, the amino group and a variable (R) group [35,36]. Three model examples have been selected, namely, glutamic acid, tyrosine, and phenylalanine. The analytes used herein form a double hydrogen-bonded complex with the CSP carbamate group. The protonated amino group of the analytes and the carbonyl group of the CSP form hydrogen bonds with the CSP carbonyl and amide groups.

Glutamic acid expressed Rs of 1.71 and α of 1.65 under standard mobile phase composition on Chiralpak^®^ ID-U (Figure 6). This is opposed to the weaker stereoselective results obtained with Chiralpak^®^ IG-U under different standard mobile phase compositions. Glutamic acid is an acidic compound with a hydrogen acceptor atom in its side chain which is negatively charged. It is very polar and can easily engage in ionic bonds through electrostatic attractions [23]. Similarly, tyrosine has both a hydrogen donor and acceptor atoms in its side chain [35,36,37,38,39,40]. Its hydroxyl group is considered uncharged and can engage in hydrogen bonds [41]. The polarity of glutamic acid and tyrosine could explain the reasons for the unsuccessful separation using [amylose tris (3-chloro-5-methylphenylcarbamate)] or Chiralpak^®^ IG-U, since it exhibits a hydrophobic methyl group.

Conversely, phenylalanine has no hydrogen donor or acceptor atoms in its side chain [38,39,40,41], whereas Chiralpak^®^ IG-U has both a methyl and chloro group. This could explain the poor stereoselectivity of Chiralpak^®^ ID-U compared to Chiralpak^®^ IG-U under different mobile phase conditions with the best Rs of 1.83 and α of 1.63 under *n*-hexane/EtOH (80/20, *v/v*) and the lowest Rs of 0.92 and α of 1.40 under MtBE/EtOH (98/2, *v/v*) (Figure 6).

### 3.4. Effect of Polar Solvents on Enantioselectivity

Apart from the standard mobile phase compositions used (alkane/alcohol modifier), the literature reveals that ACN and MtBE, together with the standard solvents, are those with the highest potential in terms of enantioselectivity [42]. Starting with non-standard organic solvents in the mobile phase composition, neat ACN and MtBE (100%, *v*) were investigated as eluents for enantioselective separation.

#### 3.4.1. Acetonitrile

ACN has unique characteristics such as its ability to dissolve a wide range of solutes, low acidity, minimal chemical reactivity, low UV cut-off, and low viscosity. The unique properties of ACN render it the solvent of choice in the separation of pharmaceuticals. However, since ACN is a poor hydrogen bonding solvent, chiral compounds analyzed with large amounts of ACN can form hydrogen bonds with the CSP [23,24,43]. Contrary to our expectations, a large percentage of compounds were separated under neat ACN (100, *v*), though RT was decreased. Of particular interest is that antifungals used in this study were only separated under ACN (100, *v*) using Chiralpak^®^ IG-U, with sulconazole expressing Rs of 1.49 and α of 1.57 and miconazole expressing Rs of 2.00 and α of 1.92.

The addition of water to ACN enhanced Rs at the expense of a longer RT for all tested analytes herein. These results were consistent with a reversed phase mechanism, where the addition of water weakened the mobile phase strength, and RT increased [42]. For example, 6-hydroxyflavanone enhanced Rs from 1.30 to 1.87 and α from 1.24 to 2.57 on Chiralpak^®^ IG-U. On the other hand, the addition of water to ACN decreased Rs from 1.68 to 1.53 and α from 3.70 to 3.02 on Chiralpak^®^ ID-U. Additionally, the use of neat ACN (100, *v*) improved the peak shape on Chiralpak^®^ ID-U (Figure 7).

#### 3.4.2. Methyl tert Butyl Ether (MtBE)

Apart from the alkanes, MtBE has the weakest eluting strength among the solvents investigated in this study. Therefore, it is possible to use it in its pure form. Neat MtBE (100, *v*) showed an enhanced Rs and α under Chiralpak^®^ IG-U for compounds such as nomifensine, normetanephrine, and epinephrine. For example, nomifensine showed Rs of 4.08 and α of 3.86 under MtBE (100, *v*) compared to Rs of 1.78 and α of 2.41 under *n*-hexane/EtOH (80/20, *v/v*). However, it has been proven that neat MtBE (100, *v*) may sometimes not be strong enough for compounds eluted within a reasonable time length and the peak shape is poor: broad peaks with large tailing have been previously observed [42].

Several solvents with higher eluting strength, such as EtOH, ACN, and 1,4-dioxane, can be efficiently used as modifiers in MtBE to improve separations [42]. It should be noted that the modifier providing the best separation results depend on the compound to be resolved [43]. Although the percentage of a modifier is generally low (mostly 2–10% in MtBE), its nature can greatly affect the enantioselectivity of a given compound. For example, the addition of 5% EtOH can reduce RT by half and the peak shape is significantly improved without deteriorating the selectivity [42].

For example, in Chiralpak^®^ IG-U, 6-hydroxyflavanone under MtBE/EtOH, (95/5, *v/v*) resulted in Rs of 0.46 and α of 1.22. The substitution of EtOH with 5% ACN enhanced Rs up to 2.69 and α to 3.69 and resulted in better peak shape. The best Rs and α values were eventually achieved with 10% 1,4-dioxane as a modifier. On the other hand, for the same compound under Chiralpak^®^ ID-U, the lowest Rs and α values were achieved under 10% 1,4-dioxane in MtBE. Five percent EtOH resulted in Rs of 12.12 and α of 4.04. This was further enhanced to Rs of 15.47 and α of 5.90 when ACN was substituted with EtOH. Of particular interest was that compounds such as tocainide, ifosfamide, and amino glutethimide were only separated under MtBE with an organic modifier (2–10%) using Chiralpak^®^ ID-U.

## 4. Conclusions

In this work, the influence of mobile phase composition on the stereoselectivity of enantiomers was studied on two sub-2 µm columns. Regarding the two non-polar solvents (alkanes) containing a polar alcohol modifier (EtOH, 2-PrOH, and *n*-BuOH), EtOH expressed the best enantioselectivity on the two CSPs. In particular cases, 2-PrOH fit better on Chiralpak^®^ ID-U. For the non-standard solvents (MtBE with organic modifiers), Chiralpak^®^ IG-U expressed the best enantioselectivity using 10% 1,4-dioxane, while 10% 1,4-dioxane was not sufficient on Chiralpak^®^ ID-U. The use of aqueous solutions such as ACN in water enhanced enantioselectivity of all racemates compared to similar separations using neat ACN.

Twenty-seven compounds were baseline/partially separated on Chiralpak^®^ IG-U compared to 22 compounds separated on Chiralpak^®^ ID-U. Chiralpak^®^ IG-U separated compounds that were not separated under any mobile phase composition on Chiralpak^®^ ID-U, namely, cizolirtine, naftopidil, sulconazole, miconazole, 1-phenyl-2,2,2-trifluoroethanol, and phenylalanine. In conclusion, mobile phase composition, the structure of the analytes, and their interaction with the CSP all play a role in enantioselectivity.
